# Comparing the Effect of Metformin and Acarbose Accompanying Clomiphene on the Successful Ovulation Induction in Infertile Women with Polycystic Ovary Syndrome

**DOI:** 10.5539/gjhs.v8n9p281

**Published:** 2016-01-31

**Authors:** Masomeh Rezai, Mohmmad Jamshidi, Robabeh Mohammadbeigi, Fariba Seyedoshohadaei, Somaye Mohammadipour, Ghobad Moradi

**Affiliations:** 1 Department of Obstetrics & Gynecology, Faculty of Medicine, Kurdistan University of Medical Sciences, Sanandaj, Iran; 2 Cellular and Molecular Research Center, Zahedan University of Medical Sciences, Zahedan, Iran; 3 Department of Obstetrics & Gynecology, Faculty of Medicine, Iran University of Medical Sciences, Tehran, Iran; 4 Social Determinants of Health Research Center, Kurdistan University of Medical Sciences, Sanandaj, Iran

**Keywords:** acarbose, infertility, metformin, ovulation induction, Polycystic Ovary syndrome

## Abstract

The aim of this study was to compare the effects of Metformin and Acarbose accompanying Clomiphene on the successful ovulation induction in infertile women with polycystic ovary syndrome. This randomized double blind clinical trial study was performed on 60 women with polycystic ovary syndrome. Women were selected and randomly divided in two control and intervention groups. Intervention group received Acarbose 100 mg/day for 3 months. In the first, second, and third weeks, they received 1 tablet, 2 tablets, and 3 tablets per day respectively. In addition, they received 100 mg Clomiphene from third to seventh day of menstruation, during the 3 month treatment period. The control group received Metformin 500 mg/day for 3 months. In the first, second, and third weeks, they received 1 tablet, 2 tablets, and 3 tablets per day respectively. In addition, they received 100 mg Clomiphene from third to seventh day of menstruation, during the 3 month treatment period. All the subjects in both groups before and after the treatment were examined for hirsutism, acne, oral glucose tolerance test, serum triglycerides, cholesterol, LDL, HDL. Also, induction of ovulation was assessed by vaginal ultrasound. The Mean of BMI and fasting glucose tolerance test in Acarbose group was less than Metformin group (P = 0.05). The mean of triglycerides, LDL and HDL levels did not differ between the two groups after the intervention (P > 0.05). The mean of cholesterol levels were different in the two groups after the intervention (P = 0.04). Frequency of ovulation induction in those who received Acarbose (78.5%) was more than those who received Metformin (46.6) (P = 0.012). Comparing with Metformin, Acarbose accompanying Clomiphene was more effective in ovulation induction and decreasing body mass index in infertile women with polycystic ovary syndrome.

## 1. Introduction

Polycystic Ovary syndrome (PCOS) is a heterogeneous endocrine disorder. One out of every 15 women (approximately 5-10% of women) is suffering from it globally. The basic disorder in this syndrome is increasing androgen secretion and in many patients it is due to abnormal insulin activity. The cause of this syndrome is unknown, but genetic studies considered the impact of prenatal environment, lifestyle or both (Sonez, 2005). Most women with PCOS and infertility are insulin resistant. High secretion of insulin may stimulate ovarian androgen secretion and a decrease in insulin production may decrease ovarian androgen secretion and increase ovulation rate (Galluzzo, Amato, & Giordano, 2008).

Some studies have shown that the combination of Metformin and Clomiphene for ovulation induction in women with PCOS is more effective than Clomiphene alone (Leanza et al., 2014) other studies have also shown that in women, who received Metformin, pregnancy rate was higher and abortion rate was lower (Moll et al., 2008). However, Moll and Legro studies showed that adding Metformin to Clomiphene did not increase the live birth rate (Moll et al., 2008; Legro et al., 2007). Acarbose is a glucosidase inhibitor, which is used in the treatment of type 2 diabetes and is also under study in Clomiphene-resistant patients (Karimzadeh, & Javedani, 2010). Other studies have shown that Acarbose improve hirsutism, acne, and menstrual irregularities through a reduction in the increasing levels of androgens and androgen band. In comparison, Acarbose reduces more weight and improve menstrual irregularities and signs of fertility in Clomiphene-resistant women with PCOS. In obese women with PCOS using Acarbose, symptoms of cardiovascular risk were decreased significantly after 6 months of treatment. Also, gastrointestinal complications in patients who took Acarbose are less than patients who took Metformin (Kircher, & Smith, 2008).

Given the contradiction of the effects of Acarbose compared with Metformin on the clinical symptoms and induced ovulation in women with polycystic ovary, the aim of this study was to compare the effects of Metformin and Acarbose accompanying Clomiphene on the successful ovulation induction in infertile women due to polycystic ovary syndrome.

## 2. Method

This double-blind clinical trial study was conducted on infertile women with polycystic ovary syndrome who referred to Besat Hospital clinic, Sanandaj, Iran in 2014. The Inclusion criteria were; 20 to 40 years of age, Rotterdam standard diagnostic criteria A: oligomenorrhea or amenorrhea, B: clinical findings or biochemical hyperandrogenism and C: polycystic ovaries on ultrasound. Exclusion criteria were: smoking, Cushing’s syndrome, thyroid dysfunction, tumors that generate androgens; patients with liver, kidney and heart disease, diabetes; treatment with other drugs of polycystic ovary syndrome such as Aldactone, Diane and Clomiphene and finally Clomiphene- resistant patients. The sample size was 60 patients who randomly divided into intervention and control groups using 4 blocking methods. This study was approved by the Ethics Committee of Kurdistan University of Medical Sciences and has been registered in the Iranian Registry of Clinical Trials with registration number IRCT2014092912789N7.

Infertile women who were willing to participate in the study and had inclusion criteria were enrolled in the study. Written consent was obtained from the participants. The Intervention group received Acarbose 100 mg/day for 3 months. In the first, second, and third weeks, they received 1 tablet, 2 tablets, and 3 tablets per day respectively. In addition, they received a daily dose of 100 mg Clomiphene for 3 months during the treatment period. The control group received Metformin 500 mg/day for 3 months. In the first, second, and third weeks, they received 1 tablet, 2 tablets, and 3 tablets per day respectively. Also, they received 100 mg Clomiphene daily during the 3 months treatment period. All the subjects in both groups before and after the treatment were examined for hirsutism, acne, oral glucose tolerance test, serum triglycerides, cholesterol, LDL and HDL. Also, induction of ovulation was assessed using vaginal ultrasound.

A gynecology resident followed up the patients during the course of treatment for drug usage and possible side effects, including gastrointestinal side effects such as bloating and diarrhea. For blinding purposes, Acarbose and Metformin were given to patients in the same packages. Also, drug packages were designed as A and B and a person who gave the drugs to the patients were unaware of the contents of the packages.

Data of 58 women participated in the study were entered into SPSS version 18 (2 patients in Acarbose group withdrew from the study due to diarrhea and gastritis). Descriptive statistics (frequency, mean and standard deviation) as well as chi-square, t-test and Fisher’s test were used to analyze the data.

## 3. Results

Results of this study showed that the mean age of Metformin group was 4.7 ± 26.3 and in Acarbose group it was 4.6 ± 26.3 years. In terms of education, 75% of Acarbose and 70.5% of Metformin group had a high school diploma or less. 80% of Acarbose group and 86.6% of Metformin group were housewives. 30% of the Metformin group and 21.55% of Acarbose group were from rural areas.

Duration of infertility and PCOS in Metformin group was 2.66 ± 1.66 years and 47.2 ± 31.9 months and in Acarbose group they were 3.67 ± 6.52 years and 44.6 ± 22.1 months respectively. In terms of previous treatment history, 50% of the Acarbose group and 70% of Metformin group had a history of treatment.

Mean of BMI and fasting glucose tolerance test in Acarbose group was less than Metformin group (P = 0.05). The mean of triglycerides, LDL and HDL levels did not differ between the two groups after the intervention (P > 0.05). The mean of cholesterol levels were different in the two groups after the intervention (P = 0.04) (Table 1). Frequency of ovulation induction in those who received Acarbose (78.5%) was more than those who received Metformin (46.6) (P = 0.012). Frequency of hirsutism and acne in two groups were not different (Table 2), also frequency of gastrointestinal side effects was similar in both groups (P > 0.05) (Table 3).

**Table 1:** Comparison of the clinical and laboratory findings before and after treatment

Clinical Tests	Before			After		
	
	Acarbose	Metformin	P Value	Acarbose	Metformin	P Value
Fasting glucose tolerance test	88.2 ± 16.o5	92.1 ± 16.3	0.37	83.3 ± 10.7	93.6 ± 19.3	0.024
glucose tolerance test 2 hours after	125.6 ± 25.9	129.7 ± 24.2	0.61	122 ± 15.6	127.3 ± 16.2	0.23
Triglycerides	122.8 ± 42.3	128.2 ± 47.7	0.65	118.1 ± 36.5	123.5 ± 31	0.56
Cholesterol	173.6 ± 28.6	183.9 ± 25.5	0.15	162.2 ± 36.5	176.9 ± 31	0.04
LDL	100.4 ± 27.8	109.2 ± 24.6	0.21	93.3 ± 20.3	103.6 ± 26.5	0.12
HDL	45.4 ± 5.7	46.3 ± 12.9	0.74	44.3 ± 7.6	47.4 ± 12.2	0.27
BMI	26.9 ± 1.8	27.3 ± 2.4	0.28	25.9 ± 1.9	27.2 ± 2.4	0.04

Results are expressed as mean ± SD.

**Table 2:** Comparison of the clinical findings before and after the treatment

	Before			After		
	
	Acarbose	Metformin	P	Acarbose	Metformin	P
ovulation induction						0.012
Yes	0	0		22 (78.5)	14 (46.6)	
No	28 (100)	30 (100)		6 (22.5)	16 (53.4)	

Acne						0.11
Yes	9 (32)	8 (36.7)	0.64	0	2 (25)	
No	19 (68)	22 (73.3)		9 (100)	6 (75)	

Menstrual disorder						0.15
Yes	23 (82.1)	24 (80)	0.83	17 (73.9)	13 (54.1)	
No	5 (17.9)	6 (20)		6 (26.1)	11 (45.9)	

Hirsutism						0.99
Yes	14 (50)	14 (46.6)	0.8	2 (14.2)	2 (12.2)	
No	14 (50)	16 (53.4)		12 (85.8)	12 (85.8)	

Data are No. (%).

**Table 3:** Comparison of the side effects in both groups

Side Effects	Group		(χ^2^)	P Value
	Metformin	Acarbose		
Distension				0.65
Yes	7 (23/3)	8 (28/6)	0.2	
No	23 (76/7)	20 (71/4)		

Vomiting				0.2
Yes	0	2 (7/1)	2.2	
No	30 (100)	26 (92/9)		

Diarrhea				0.88
Yes	8 (26.7)	7 (25)	0.02	
No	22 (73.3)	21 (75)		

Anorexia				0.65
Yes	8 (26.7)	9 (32.1)	0.21	
No	22 (73.3)	19 (67.9)		

## 4. Discussion

In recent years, oral anti-diabetic drugs such as Metformin, Troglitazone, Rositroglitazone, and Acarbose are used for the treatment of patients with insulin resistance PCOS. Metformin is a biguanide antihyperglycemic that is widely used in patients with PCOS. Studies have shown the effect of Metformin in reducing insulin and improving the metabolic status of patients (Lord et al., 2003). Metformin side effects include anorexia, diarrhea, nausea, and vomiting suggesting the decrease in the use of this medication in patients with PCOS. In addition, an increase in blood lactic acid may be a life-threatening complication in diabetic patients (Lord et al., 2003; Jeffrey Chang, 2004) although these side effects have not been reported in women with PCOS (Legro, 2007).

In this study, the mean of BMI was changed from 26.9 to 25.9 kg/m^2^ in Acarbose group and from 27.3 to 27.2 in the Metformin group. This showed a significant reduction of body mass index in Acarbose group. Weight loss in patients with type II diabetes after taking Acarbose has been reported in other studies (Chiasson et al., 2002). Kircher’s systematic review has confirmed weight loss in women using Acarbose (Kircher, 2007). In Sönmez study, the mean of BMI in Acarbose group before treatment was 27.4 kg/m^2^ and after treatment it was 26.3 kg/m^2^. Mean of BMI in the Metformin group before and after treatment were 27.2 and 26.9, respectively, and there were significant differences between the two groups (Sonmez, 2005). In a study by Ciotta et al., Acarbose was used in 30 PCOS patients with normal BMI. In this group, there was no significant change in body mass index (Ciotta et al., 2001). In the same study, 61 women with PCOS were treated with Metformin and there were no significant changes in BMI (Nestler et al., 2002). In the meta-analysis conducted by Zhang et al. in 2014, to investigate the effects of Acarbose in patients with PCOS, there were no changes in the body mass index (Zhang, Hou, & Zhao, 2014). Therefore; our findings are consistent with most of the above similar studies.

In the small intestine, Acarbose creates a dose-dependent reversible bond with alpha-glucosidase position in Oligosaccharides that reduces oligosaccharides and disaccharides hydrolysis, leading to reduced absorption of monosaccharide in the blood stream. Acarbose reduces 20% after meal blood sugar and increases glucose uptake, this will prevent the Hyperinsulinemia. This drug, through reducing absorption of glucose, leads to an indirect increase in Glucagon-like peptide-1 and by affecting the satiety center of the brain reduces appetite and weight (Qiao et al., 2015).

The prevalence of hirsutism before and after the treatment was not significantly different in the two groups and improved patients were similar in both groups. In the meta-analysis conducted by Zhang et al in 2014, the Galway Freeman criteria were not changed (Zhang, Hou, & Zhao, 2014). Ciotta showed favorable impression of Acarbose on Freeman Galway criteria (Ciotta et al., 2001). In a study by Penna, Galway Freeman criteria in Acarbose group were lower than the control group. Given that incidence of hirsutism in the control group was higher before treatment, this difference was not necessarily related to the effects of Acarbose (Penna, 2005). Kircher approved hirsutism improvement in women using Acarbose (Kircher, 2008). Because of the inverse relationship between weight and sex hormone binding globulin, Acarbose increases this globulin through weight loss and increases insulin sensitivity and decreases LH and hyperandrogenism (Ciotta et al., 2001). Probably the cause of this difference is related to the duration of the intervention or treatment with Acarbose. Given the short period of 3 months of treatment in this study and long-term treatment of hirsutism, failure in treatment is logical.

In the present study, the mean of fasting glucose tolerance test was reduced after the intervention in Acarbose group than before, and had significant difference with Metformin group. In some studies Metformin 300 mg daily with Acarbose 1.5 gr daily in PCOS patients, regardless of the weight was investigated and metabolic parameters were similar (Sonmez, 2005; Willson, 2008; Barbeiri & Ehrmann, 2010; Ehrman, 2005; Azziz, 2009). Hanjalic-Beck showed that only in patients who had been treated with Metformin, levels of fasting insulin reduced significantly (Hanjalic-Beck et al., 2010). Ciotta showed desired effects of Acarbose on insulin sensitivity improvement (Ciotta, 2001). A study showed that Acarbose did not cause hypoglycemia or lactic acidosis (Guilherme et al., 2008). This approach strengthened using a combination of these two drugs.

In both groups lipid profile reduced after the intervention compared to the before, but cholesterol level in Acarbose group was significantly lower than Metformin group. In Hanjalic-Beck study, patients who had been treated with Metformin showed significant reduction in cholesterol levels (Hanjalic-Beck et al., 2010). In Zhang et al meta-analysis, they showed that Acarbose reduces triglycerides and LDL and increases HDL compared with placebo (Zhang, Hou, & Zhao, 2014). The results showed that improvement in menstrual disorders of Acarbose group (73.9%) was greater than Metformin group (54.1%). Other studies, including Hanjalic and Beck study the prevalence of menstrual regulation in the Metformin group were 70% and in Acarbose it was 78% (Hanjalic-Beck et al., 2010). Penna’s study showed that in patients who received Acarbose compared to the control group, they had 2.67 times regular menstrual cycles (Penna, 2005). In Ciotta’s study, 60% of patients in Acarbose group had regular menstrual periods (Ciotta, 2001). Kircher’s systematic review confirmed menstrual cycle improvement in women who used Acarbose (Kricher, 2008). In Zhang’s study, there was no significant difference between Acarbose and Metformin regarding menstrual pattern (Zhang, Hou, & Zhao, 2014). The results of most of the studies are consistent with our findings. It seems that menstrual regulation is associated with weight loss, secretion of SHBG, free androgen reduction, and performance of ovarian hormones (Penna, 2005).

In our study, there was no difference between the two groups regarding acne treatment. But, Metformin group was better than Acarbose. Ciotta showed favorable effects of Acarbose on acne (Ciotta, 2001). In his systematic review, Kricher showed a positive effect of Acarbose on acne (Kricher, 2008).

Frequency of ovulation induction in Acarbose group (78.5%) was significantly higher than Metformin group (46.6%). In Ciotta’s study, there was not a method to determine the ovulation rate during treatment with Acarbose (Ciotta, 2001). In Zhang et al meta-analysis, there was no significant difference between Acarbose and Metformin groups regarding increases in the ovulation rate (Zhang, Hou, & Zhao, 2014). Sönmez et al showed that the ovulation rate increased in both Acarbose and Metformin groups and indicated that administration of Metformin improved follicle response to gonadotropins (Snomez, 2005). In 2010 Hanjalic-Beck studied the effects of Metformin versus Acarbose on ovulation rate. They studied the impact of hormones and metabolism in patients with polycystic ovary syndrome (PCOS). The ovulation rate in the Metformin group was 73% and in Acarbose was it was 59%, but the difference was not statistically significant (Hanjalic-Beck et al., 2010).

Frequency of gastrointestinal side effects, including distension, vomiting, diarrhea and anorexia in both groups (about 28%) was the same. In Zhang et al study, Acarbose caused side effects such as abdominal pain and diarrhea (OR = 23.78) (Zhang, Hou, & Zhao, 2014). In another study, mild complications were observed in 84% of the patients in Acarbose group (Penna, 2005). In Hanjalic study, in Acarbose group (38%) distension and diarrhea was less compared with Metformin group (80%) (Hanjalic-Beck et al., 2010). Acarbose intestinal absorption rate was less than Metformin (1.7% - 5.0%) and this property reduced the dose dependent side effects such as distension and diarrhea. These side effects were minimized with Acarbose gradual increase. The most important Metformin side effects were nausea, vomiting, fatigue, diarrhea, particularly in patients with chronic renal failure. Also, Metformin reduced the intestinal absorption of vitamin B12. Due to the incidence of side effects, choosing the proper treatment method could be a limiting factor; therefore, Acarbose appears to be safer and more effective in the treatment of PCOS (Ciotta, 2001).

Penna et al study suggested using a combination of drugs for patients with PCOS, particularly in obese patients that standard treatments are less effective (Penna, 2005). Although there are a lot about clinical, metabolic, and reproductive benefits of Metformin; however, stabilizing the controlled studies showed that the results were moderate (Harborne et al., 2003). This has opened a new horizon in the combination therapy.

In conclusion, regarding ovulation induction and reduction of body mass index of women with PCOS, usage of Acarbose with Clomiphene is more effective than Metformin. Also Acarbose reduces the lipid profile better than Metformin; though, they have the same gastrointestinal side effects.
